# Determination of time-varying pressure field from phase contrast MRI data

**DOI:** 10.1186/1532-429X-14-S1-W36

**Published:** 2012-02-01

**Authors:** Lucian M Itu, Puneet Sharma, Mehmet A Gulsun, Viorel Mihalef, Ali Kamen, Andreas Greiser

**Affiliations:** 1 Image Analytics and Informatics, Siemens Corporate Research, Princeton, NJ, USA; 2 Siemens AG, Erlangen, Germany

## Background

The aim of this work is to investigate the feasibility of determining patient-specific time-varying pressure distribution along the vessel centerlines in 4D PC-MRI data.

## Methods

**Figure 1:**
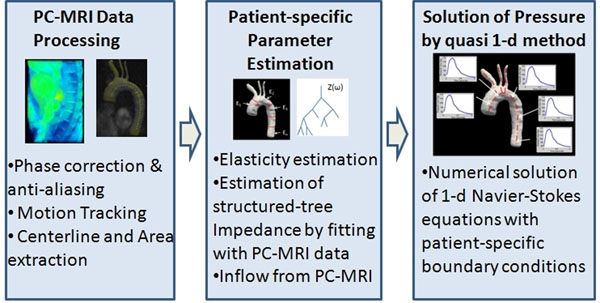
Overview of the proposed methodology

We propose a numerical framework for determining pressure field in large arteries, the main components of which are: axi-symmetric 1-D unsteady wave-propagation model with elastic walls and an optimization framework for model personalization via parameter estimation from measured flow and anatomical data (Fig 1).

4D PC MRI data was acquired using a multi-slice phase contrast FLASH cine sequence with 3d velocity encoding (Siemens Magnetom Verio, temporal resolution 52 ms, matrix 100x192, FOV 319x219 mm, slice thickness 5 mm) in a sagittal to coronal slice orientation. Siemens 4D Flow WIP software was used for background phase, motion and venc anti-aliasing corrections, extracting vessel models including centerline trees and lumen boundary, and for automated extraction of flow profiles and cross-sectional areas.

We use a linear elastic model for the vessel wall, with an axially varying stiffness. The parameters of the material model are estimated by a numerical optimization procedure, wherein the simulated deformation is fitted to the observed deformation of the vessel wall extracted from the MR data. Unsteady flow rate at the aortic inlet is extracted from PC-MRI data and used as the inlet boundary condition. To obtain physiologically valid mean and pulse pressure values and the wave propagation effects, we use a structured tree outflow boundary condition, wherein a morphological or fractal vessel tree is introduced to model the downstream behavior at the outlets of the vessel tree. The parameters for the outlet boundary condition are then independently perturbed at each individual outlet in order to match the measured unsteady outflow rate from PC-MRI data. This is achieved by adapting the final radius at which the asymmetric binary tree is terminated.

The hyperbolic PDEs are solved using finite differences based on 2nd-order Lax-Wendroff method. Simulations converge after 3 cycles, with a maximum relative difference of 1% in pressure and flow between 2 consecutive cycles.

## Results

**Figure 2:**
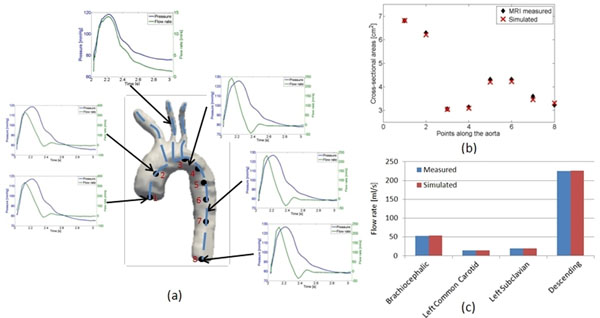
(a) Pressure and Flow rate distribution along the vessel centerline tree. The blue curve represents the pressure, while the green curve represents the flow rate. The results have been plotted for the 3rd cardiac cycle. (b) Comparison of the measured and simulated cross-sectional areas along the centerline at peak systole. (c) Comparison of the measured and the simulated flow rates in the Brachiocephalic, Left Common Carotid, Left Subclavian, Descending Aorta during peak systole.

The computed pressure distribution along the centerline is shown in Fig 2a. Flow rates and cross-sectional area variation are in good agreement with the PC-MRI data (Fig 2b, 2c), reiterating the patient-specific nature of the proposed method. Flow rates in the 4 branches differed in the range of 1%-4% when compared to PC-MRI data. A physiological pressure waveform with a pulse pressure approximately equal to 45 mm Hg was obtained.

## Conclusions

We presented a technique for the determining pressure field from PC-MRI data in large vessels. The method is being extended for diseased cases (stenosis, aneurysms etc) by coupling empirical models in the arterial tree to account for the pressure changes at specific locations. Further, sudden changes in wall distensibility, like those in stented regions can be modeled directly.

